# Neuropsychiatric or Behavioral and Psychological Symptoms of Dementia (BPSD): Focus on Prevalence and Natural History in Alzheimer's Disease and Frontotemporal Dementia

**DOI:** 10.3389/fneur.2022.832199

**Published:** 2022-06-24

**Authors:** Valentina Laganà, Francesco Bruno, Natalia Altomari, Giulia Bruni, Nicoletta Smirne, Sabrina Curcio, Maria Mirabelli, Rosanna Colao, Gianfranco Puccio, Francesca Frangipane, Chiara Cupidi, Giusy Torchia, Gabriella Muraca, Antonio Malvaso, Desirèe Addesi, Alberto Montesanto, Raffaele Di Lorenzo, Amalia Cecilia Bruni, Raffaele Maletta

**Affiliations:** ^1^Association for Neurogenetic Research (ARN), Lamezia Terme, Italy; ^2^Regional Neurogenetic Centre - ASP-CZ, Lamezia Terme, Italy; ^3^Department of Mathematics and Computer Science, University of Calabria, Rende, Italy; ^4^Neurology Unit, Fondazione Istituto Giglio, Cefalù, Italy; ^5^Neurology Unit, IRCCS San Raffaele Scientific Institute, Milan, Italy; ^6^Department of Internal Medicine, Pugliese Ciaccio Hospital, Catanzaro, Italy; ^7^Department of Biology, Ecology and Earth Sciences, University of Calabria, Rende, Italy

**Keywords:** behavioral and psychological symptoms of dementia, BPSD, Alzheimer's disease, frontotemporal dementia, prevalence, natural history, neuropsychiatric profile, psychotic symptoms

## Abstract

Neuropsychiatric or behavioral and psychological symptoms of dementia (BPSD) represent a heterogeneous group of non-cognitive symptoms that are virtually present in all patients during the course of their disease. The aim of this study is to examine the prevalence and natural history of BPSD in a large cohort of patients with behavioral variant of frontotemporal dementia (bvFTD) and Alzheimer's disease (AD) in three stages: (i) pre-T0 (before the onset of the disease); (ii) T0 or manifested disease (from the onset to 5 years); (iii) T1 or advanced (from 5 years onwards). Six hundred seventy-four clinical records of patients with bvFTD and 1925 with AD, from 2006 to 2018, were studied. Symptoms have been extracted from Neuropsychiatric Inventory (NPI) and from a checklist of BPSD for all periods observed. In our population, BPSD affect up to 90% of all dementia subjects over the course of their illness. BPSD profiles of the two dementia groups were similar but not identical. The most represented symptoms were apathy, irritability/affective lability, and agitation/aggression. Considering the order of appearance of neuropsychiatric symptoms in AD and bvFTD, mood disorders (depression, anxiety) come first than the other BPSD, with the same prevalence. This means that they could be an important “red flag” in detection of dementia. With the increase of disease severity, aberrant motor behavior and wandering were significantly more present in both groups. Differences between BPSD in AD and bvFTD resulted only in prevalence: Systematically, in bvFTD, all the symptoms were more represented than in AD, except for hallucinations, depression, anxiety, and irritability. Given their high frequency and impact on management and overall health care resources, BPSD should not be underestimated and considered as an additional important diagnostic and therapeutic target both in patients with AD and bvFTD.

## Introduction

Dementia is an insidious syndrome characterized by the progressive decline of mental functions. In addition to cognitive deficits, at least 90% of patients exhibit various neuropsychiatric or behavioral and psychological symptoms of dementia (BPSD) at any given point in the course of their illness (1). BPSD can be defined as a wide range of non-cognitive symptoms involving perception (e.g., hallucinations), mood (e.g., depression, anxiety), behavior (e.g., aggression, disinhibition), personality, and basic functioning (2–6). The pathogenesis of these symptoms is not still completely understood, and the current knowledge supports multifactorial causes, involving the interaction of biological, psychological, and social factors (7). It has been shown that BPSD are associated with high levels of distress both in dementia sufferers and their caregivers, as well as with adverse outcomes, long-term hospitalization, and misuse of medications and increased use of health care resources (8–12). Thus, in addition to cognitive deterioration, BPSD are a relevant and meaningful clinical target for intervention (13) although are still now considered as non-disease–specific markers (14). Despite the importance of these symptoms in both clinical characterization and prognosis of dementia, the few studies performed on their natural history and prevalence achieved mixed results (15–20). However, previous studies suggest that some of these symptoms are more predominant in one type of dementia than in another (15). Instead, understanding the full course of BPSD in different forms of dementia might be important to guide treatment choices and to improve the quality of life of both patients and their caregivers. In particular, we decided to focus our attention on the comparison of prevalence and natural history of BPSD between Alzheimer's disease—the most prevalent form of senile dementia (21)—and the behavioral variant of Fontotemporal Dementia—the most common form of presenile dementia (16).

### Alzheimer's Disease (AD) and BPSD

Alzheimer's disease (AD) is the most widespread neurodegenerative disorder worldwide (20–22), typically characterized by memory loss and other cognitive deficits (20, 23). BPSD are being increasingly recognized as common serious problems in AD (8). Several findings suggest that some BPSD—i.e., depression, anxiety, and apathy—can occur before the onset of cognitive decline in AD (8, 24, 25) and could predict both cognitive decline and progression from MCI to AD (26). On the other hand, it has been reported an onset of BPSD several months after AD diagnosis (27). Conflicting results were also obtained about the prevalence of BPSD. Several findings showed that apathy is the most common BPSD in AD (20, 28, 29). Conversely, other studies indicated sleep disturbances (17), irritability/aggression (18), or depression (30) as the most prevalent BPSD in AD. These discordant findings stress the urgency to better characterize the prevalence and natural history of BPSD in AD.

### Frontotemporal Dementia (FTD) and BPSD

Frontotemporal dementia (FTD) is the most frequent neurodegenerative disorder with a presenile onset (16, 31). Based on clinical presentation, there are three main clinical syndromes of FTD: (i) behavioral variant FTD (bvFTD); (ii) non-fluent primary progressive aphasia (PPA); (iii) and semantic variant primary progressive aphasia (svPPA) (32). bvFTD represents the most frequent clinical picture of FTD (33) and is the only neurodegenerative disorder that requires the presence of behavioral changes to establish the diagnosis (32). These symptoms include disinhibition, apathy, hyperorality, and dietary changes (34). However, it has been shown that most people with FTD also show psychotic symptoms, such as delusions or hallucinations (35), resembling schizophrenia, major depressive and bipolar spectrum disorders (16, 36). On the other hand, Mendez et al. (19) documented an infrequency of psychotic symptoms in patients with bvFTD. Compared to patients with AD, Mukherjee et al. (15) reported a higher frequency of delusions and a low frequency of hallucinations (15), whereas Mendez et al. (37), a lower frequency of both delusions and hallucinations in bvFTD. These contradictory findings highlight the need to better understand the prevalence and natural history of BPSD in bvFTD and to make a comparison with patients with AD, in particular for psychotic symptoms.

## The Current Study

The aim of this retrospective study was to examine the prevalence and to compare the natural history of BPSD in a large cohort of patients with AD and bvFTD. More specifically, we wished to establish the frequency and type of BPSD in relation to the onset of these two types of dementia.

## Materials and Methods

### Participants and Procedure

The study population included 2,599 patients with bvFTD and AD (950 men and 1,649 women), followed at the Regional Neurogenetic Centre (ASP CZ) from 2006 to 2018. Diagnosis was performed according to criteria of The Lund and Manchester Groups Englund et al. (38) and Rascovsky et al. (32), NINCDS-ADRDA criteria (39), and National Institute on Aging and Alzheimer's Association workgroup (40). Data were retrospectively extracted from the respective medical records on the basis of completeness of clinical data. Inclusion criteria were: (1) Diagnosis of probable AD or bvFTD according to the above-mentioned criteria; (2) Availability of a reliable caregiver; (3) Completeness of clinical data; (4) Patients free from pharmacologic treatments for BPSD. Exclusion criteria were: (1) Patients with a past history of psychiatric illness and/or any neurological illness that could interfere with neuropsychological tests; (2) Unavailability of a reliable caregiver; (3) Incompleteness of clinical data; (4) Known or suspected history of alcoholism or drug abuse. Most of the patients were from Southern Italy. As this paper focuses on natural course of BPSD in dementia, we identified the moment in which the BPSD appeared in the history of the disease: (i) pre-T0 (before the onset of the disease); (ii) T0 or manifested disease (from the onset to 5 years); (iii) T1 or advanced (from 5 years onwards).

#### bvFTD Cohort

The bvFTD sample included 674 patients (317 men and 357 women). Their mean age was 66.7 ± 11.1 years. The mean follow-up was 2.6 years ± 2.8, and the first clinical observation was 4.6 ± 4.3 years after the onset of the symptoms (ETOV: Elapsing Time between Onset and first Visit).

#### AD Cohort

The AD sample included 1,925 patients (1,292 women and 633 men). Their mean age was 71.5 ± 8.9 years, the mean follow-up was 3.5 ± 2.9 years, and ETOV was 3.7 ± 3.2 years.

## Measures

A detailed anamnesis and clinical history were collected to identify the first core signs experienced by the patients or observed by family that could be identified as the moment of the onset of dementia. Exhaustive information was also collected for the time preceding the onset of dementia. We also analyzed data from the following examinations that are routinely performed in our Center at the first visit and every 6 months in all patients:

◾ Mini-Mental State Examination (MMSE) (41);
◾ Clinical Dementia Rating Scale (CDR) (42);
◾ Clinical Insight Rating Scale (CIRS) (43);
◾ Activities of Daily Living (ADL) (44);
◾ Instrumental Activities of Daily Living (IADL) (45);
◾ Neuropsychiatric Inventory (NPI) (46);
◾ A checklist encompassing wandering and the same BPSD of NPI extrapolated from the patient's history collected in the medical records (8, 47).

### Statistical Analysis

All analyses were performed with SPSS statistical software 21 (SPSS Inc., Chicago, IL, USA). Descriptive statistics, frequencies, contingency coefficient test (cross tabs) were evaluated to analyze prevalence and occurrence of BPSD. For the analysis of dichotomous variables between two groups, a chi-square cross tab test was performed. The effect of sample size on the strength of the relationship was tested using ϕ. The time of the presentation of each BPSD (a continuous variable) was calculated and compared in AD and bvFTD, using the Student's *T*-test. Cohen's d was used to calculate the effect size. Statistical significance was set at <0.05.

## Results

Table 1 reports the comparison of the demographic and clinical characteristics of the two groups (AD and bvFTD). Female gender (*p* < 0.001), late onset (*p* < 0.001), and low education (*p* < 0.001) were more represented in AD compared to the bvFTD group. Patients with AD also arrive to the first clinical consultation before patients with bvFTD (ETOV) (*p* < 0.001). On the CDR, both groups showed a slight/moderate dementia (AD < bvFTD), with patients with AD being more autonomous (on ADL and IADL, *p* < 0.001; *p* < 0.001), and more aware about their clinical status (CIRS) (*p* < 0.001) than patients with bvFTD.

**Table 1:** Baseline characteristics of the AD and bvFTD groups.

		**AD**	**bvFTD**	***p***
		**(N**°**1,925)**	**(N**°**674)**	
Age atonset, years		71.5 ± 8.9	66.7 ± 11	<0.001^**^
Gender	Female, *n* (%)	1292 (67)	357 (53)	<0.001^**^
	Male, *n* (%)	633 (33)	317 (47)	
Education	Low, *n* (%)	1,294 (68)	355 (53)	<0.001^**^
	High, *n* (%)	477 (27)	250 (37)	
Onset	Early, *n* (%)	387 (20)	256 (38)	<0.001^**^
	Late, *n* (%)	1,538 (80)	418 (62)	
Familiarity	Sporadic, *n* (%)	937 (49)	310 (46)	0.248
	Familiar, *n* (%)	988 (51)	364 (54)	
MMSE score (0–30)		16.3 ± 6.2	16.5 ± 7.2	0.710
CIRS score (0–8)		2.5 ± 2.9	4.7 ± 3.3	<0.001^**^
CDR score (0–4)		1.5 ± 0.9	1.7 ± 1.1	0.001^*^
ADL score (0–6)		4.6 ± 1.7	4.2 ± 1.9	<0.001^**^
IADL score (0–8)		3.4 ± 2.4	2.7 ± 2.4	<0.001^**^
ETOV (months)		3.7 ± 3.2	4.6 ± 4.3	<0.001^**^

*Data are presented as n (%) or mean ± standard deviation*.

* *Significant difference (p < 0.05)*.

** *Significant difference (p < 0.001)*.

### Prevalence of BPSD

**Figure 1:**
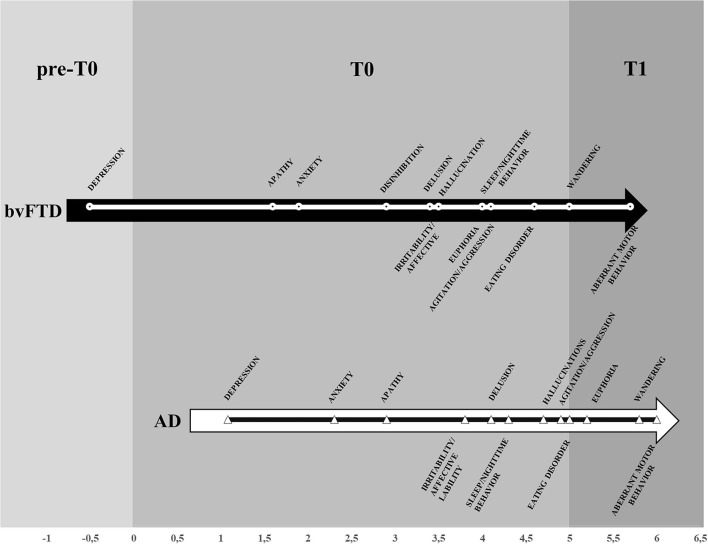
Prevalence of BPSD in AD and bvFTD groups.

The prevalence of at least one BPSD was high in both groups: 96.4% of the bvFTD sample, and 90.8% of the AD sample. The most frequent symptoms were apathy (FTD, 74.6%; AD, 57.4%), irritability/affective lability (bvFTD, 52.5%; AD, 50.5%) and agitation/aggression (bvFTD, 49.7%; AD, 42.3%) (Figure 1, Table 2). The prevalence was significantly higher in patients with bvFTD compared to patients with AD for delusions (*p* = 0.003), agitation/aggression (*p* = 0.001), euphoria (*p* < 0.001), apathy (*p* < 0.001,) disinhibition (*p* < 0.001), aberrant motor behavior (*p* < 0.001), sleep/nighttime behavior (*p* < 0.001), eating disorders (*p* < 0.001), and wandering (*p* < 0.001) (Figure 1, Table 2). T0 is the time in which BPSD were more frequent in both groups (Table 3). Euphoria and aberrant motor behavior were absent in pre-T0 in both groups. There was a difference in prevalence of BPSD between the two clinical groups in all the three periods considered. In patients with AD, the most represented BPSD in pre-T0 were depression (4.2%) and apathy (2.5%); in T0 were apathy (44.3%) and irritability/affective lability (37%); in T1 were agitation/aggression (15.9%) and irritability/affective lability (12.8%). In the bvFTD group, apathy was always the most frequent BPSD: at pre-T0 is 6.8%, followed by depression (6.7%); at T0, apathy is 62.8%, followed by irritability/affective lability (39.2%); at T1 apathy was 21.5%, followed by agitation/aggression (18.4%) (Table 3). In all three stages, each BPSD was generally more represented in bvFTD than in AD. At pre-T0, the prevalence was significantly higher in bvFTD compared to patients with AD for delusions (*p* < 0.001), hallucinations (*p* < 0.001), agitation/aggression (*p* = 0.006), euphoria (*p* < 0.001), apathy (*p* < 0.001), disinhibition (*p* < 0.001), and eating disorders (*p* = 0.003) (Table 3). At T0, for delusions (*p* = 0.001), agitation/aggression (*p* < 0.001), euphoria (*p* < 0.001), apathy (*p* < 0.001), disinhibition (*p* < 0.001), aberrant motor behavior (*p* < 0.001), eating disorders (*p* < 0.001), and wandering (*p* < 0.001). At T1 euphoria (*p* < 0.001), apathy (*p* < 0.001), disinhibition (*p* < 0.001), aberrant motor behavior (*p* < 0.001), eating disorders (*p* < 0.001), and wandering (*p* = 0.002) (Table 3).

**Table 2:** Prevalence of BPSD in the whole sample and in AD and bvFTD groups.

	**Prevalence**			***p***	**ϕ**
**BPSD**	**Total**	**AD**	**bvFTD**		
Delusions	26.4	24.8	30.7	0.003^*^	0.059
Hallucinations	28.1	27.5	29.8	0.246	0.023
Agitation/aggression	44.2	42.3	49.7	0.001^*^	0.065
Depression	39.2	38.8	40.4	0.478	0.014
Anxiety	27.2	26.8	28.5	0.400	0.017
Euphoria	4.0	2.3	9.1	<0.001^**^	0.151
Apathy	61.9	57.4	74.6	<0.001^**^	0.155
Disinhibition	29	26.3	36.9	<0.001^**^	0.103
Irritability/affective lability	51.1	50.5	52.5	0.377	0.017
Aberrant motor behavior	13.1	10.6	20.3	<0.001^**^	0.126
Sleep/Nighttime behavior	38.7	35.6	47.6	<0.001^**^	0.108
Eating disorders	20.2	13.6	39.0	<0.001^**^	0.277
Wandering	10.2	7.5	17.7	<0.001^**^	0.147

*Data are presented as %*.

* *Significant difference (p < 0.05)*.

** *Significant difference (p < 0.001)*.

**Table 3:** Prevalence of BPSD in AD and bvFTD groups divided by three periods.

	**pre-T0**				**T0**				**T1**			
**BPSD**	**AD**	**FTD**	***p***	**ϕ**	**AD**	**FTD**	***p***	**ϕ**	**AD**	**FTD**	***p***	**ϕ**
Delusions	0.4	1.9	<0.001^**^	0.078	17.2	22.8	0.001	0.064	7	9.8	0.018^**^	0.047
Hallucinations	0.3	1.9	<0.001^**^	0.083	17.5	22.1	0.009^*^	0.052	9.2	8.2	0.391	−0.017
Agitation/aggression	0.5	1.6	0.006^*^	0.058	25.7	33.7	<0.001^**^	0.078	15.9	18.4	0.136	0.030
Depression	4.2	6.7	0.013^*^	0.050	27.9	30	<0.001^**^	0.020	6.1	8.5	0.043	0.041
Anxiety	2.2	2.5	0.614	0.010	18.4	20.5	0.250	0.023	5.6	7.7	0.050	0.039
Euphoria					1.4	10.7	<0.001^**^	0.210	0.9	4.6	<0.001^**^	0.112
Apathy	2.5	6.8	<0.001^**^	0.102	44.3	62.8	<0.001^**^	0.162	10.9	21.5	<0.001^**^	0.135
Disinhibition	0.2	1.6	<0.001^**^	0.088	8.8	28.2	<0.001^**^	0.247	4.7	10.2	<0.001^**^	0.100
Irritability/Affective lability	0.7	1.8	0.018^*^	0.050	37	39.2	0.315	0.020	12.8	15.9	0.047^*^	0.040
Aberrant motor behavior					5.5	20.5	<0.001^**^	0.213	5.1	12.1	<0.001^**^	0.111
Sleep/nighttime behavior	1.2	1.8	0.270	0.022	22.3	32.8	<0.001^**^	0.106	11	14.4	0.022^**^	0.046
Eating disorders	0.1	0.7	0.003^*^	0.063	8.7	26.6	<0.001^**^	0.230	4.8	13.9	<0.001^**^	0.156
Wandering	0.1	0.1	0.468	0.15	4.0	11.7	<0.001^**^	0.142	3	5.8	0.002^*^	0.064

*Data are presented as %*.

* *Significant difference (p < 0.05)*.

** *Significant difference (p < 0.001)*.

#### Natural History of BPSD

**Figure 2:**
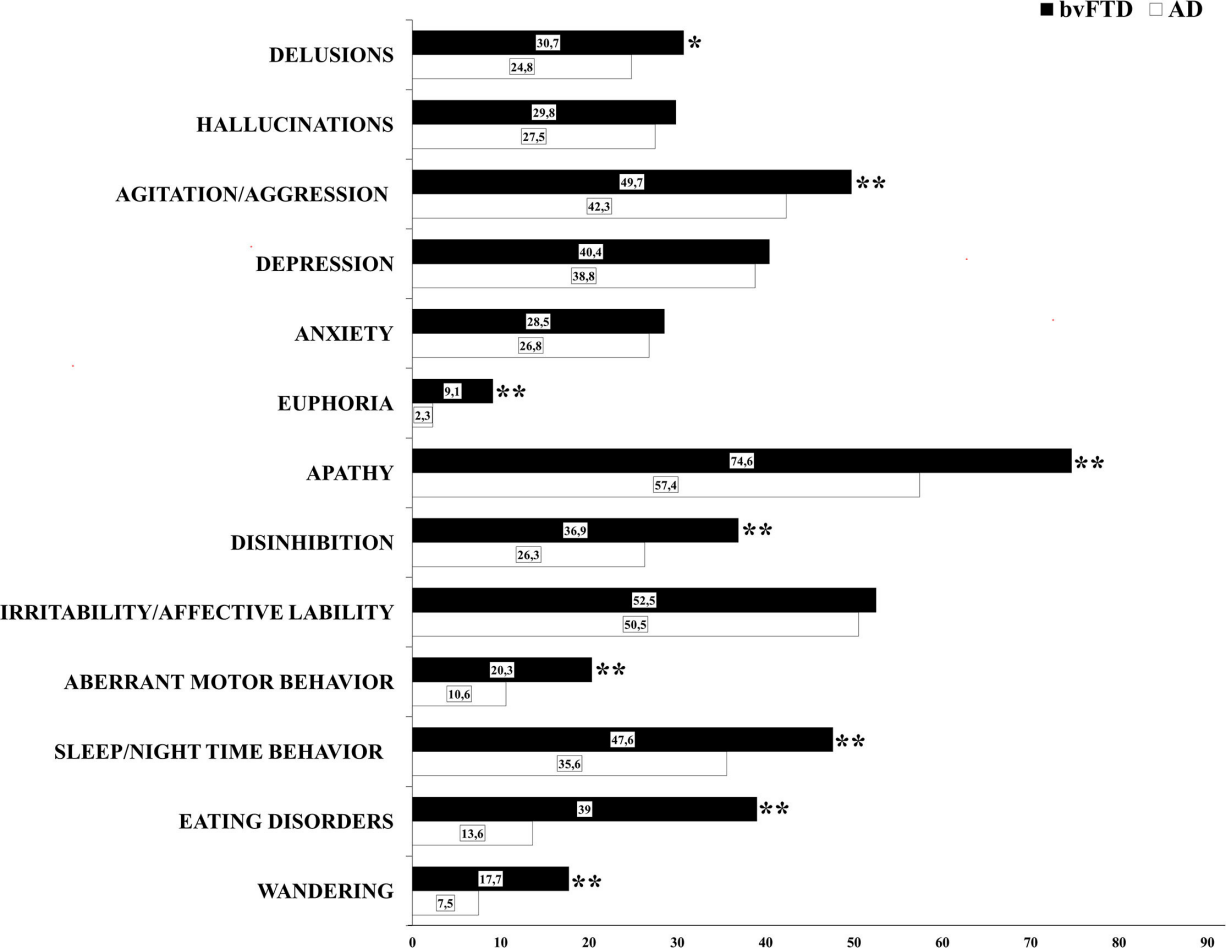
Mean of the year of the BPSD onset in the disease's history in AD and bvFTD groups. *Significant difference (*p* < 0.05). **Significant difference (*p* < 0.001).

Considering the mean (in years) of the onset of BPSD in relation to the onset of dementia, most BPSD were identified at T0, but some signs were also identified at pre-T0 and T1. Depression was the first appearing BPSD, followed by apathy and anxiety in bvFTD and anxiety and apathy in AD (Figure 2). In patients with bvFTD, depression became obvious 0.5 years before the onset of dementia, while, in AD, was reported at 1.08 years after the onset of dementia. The BPSD that significantly appear first in AD compared to patients with bvFTD were: depression (*p* = 0.002), apathy (*p* < 0.001), hallucinations (*p* = 0.001), agitation/aggression (*p* = 0.018), and disinhibition (*p* < 0.001) (Table 4, Figure 2).

**Table 4:** The BPSD onset in AD and bvFTD groups.

**Symptoms**	**AD**	**bvFTD**	***p***	**Cohen's d**
Delusions	4.1 (0.2)	3.4 (0.4)	0.124	0.15
Hallucinations	4.7 (0.2)	3.5 (0.4)	0.001^*^	0.28
Agitation/aggression	4.9 (0.2)	4.1 (0.3)	0.018^*^	0.18
Depression	1.84 (0.2)	−0.05 (0.6)	0.002^*^	0.29
Anxiety	2.3 (0.2)	1.9 (0.5)	0.415	0.08
Euphoria	5.2 (0.5)	4.0 (0.4)	0.063	0.37
Apathy	2.9 (0.1)	1.6 (0.2)	<0.001^**^	0.33
Disinhibition	5.0 (0.3)	2.9 (0.3)	<0.001^**^	0.47
Irritability/affective lability	3.8 (0.1)	3.5 (0.3)	0.249	0.09
Abberant motor behavior	5.9 (0.3)	5.7 (0.5)	0.651	0.05
Sleep/Nighttime behavior	4.3 (0.2)	4.0 (0.3)	0.406	0.06
Eating disorders	5.0 (0.3)	4.6 (0.3)	0.308	0.09
Wandering	5.8 (0.3)	5.0 (0.4)	0.125	0.19

*Data are presented as mean and standard error (in parenthesis)*.

* *Significant difference (p < 0.05)*.

** *Significant difference (p < 0.001)*.

## Discussion

BPSD are very common, nearly invariable, and are associated with high levels of distress in both dementia sufferers and their caregivers. In our study, we examined the prevalence and the timing of BPSD in a sample of 2,599 patients with AD and bvFTD. Our results confirm that, during the natural course of dementia, BPSD can be subjectively experienced by the patients and/or reported by the caregivers. We found that BPSD were present in over 90% of the whole cohort, more specifically in 96.4% in bvFTD and 90.8% in AD cohorts over the course of their illness. Apathy was the most represented symptom in both diseases (61.9% of the entire sample) in agreement with previous studies (3, 48, 49). Interestingly, in patients with bvFTD, apathy was the most frequent symptom throughout the whole course of the disease. These data are in agreement with Kumfor et al. (49) who found that 60% of patients with AD and 84% of patients with bvFTD had apathy, and it was more severe and frequent in patients with bvFTD. About patients with AD, we found that depression and apathy were the most frequent symptoms in the early phases of the disease and agitation in the late ones. These data confirm that depression and apathy could represent a wake-up call to which clinicians should pay attention for the early detection of AD (26). However, delusions and hallucinations were also present in a consistent percentage in both patients with AD and bvFTD. This is an interesting finding, as these psychotic symptoms have not been systematically reported as part of the clinical FTD (32, 50) and AD (39, 40) criteria. Based on previous studies, in general, psychotic symptoms were considered quite rare in FTD compared to other dementia diseases, and, only recently, some studies have found a higher proportion of patients with psychotic features as part of their FTD symptomatology. Landqvist Waldö et al. (35) found that psychotic symptoms were present in 32% of their FTD neuropathologically verified sample, while Legarde and Sarazin (51) found a frequency of 20%. Considering the timing of the onset of BPSD in AD and bvFTD, most of these symptoms had the onset within 5 years from the onset of dementia. However, some BPSD appeared after 5 years and, in FTD, even before the onset of dementia. Mood disorders (depression, anxiety) had an earlier onset compared to other BPSD, with the same frequency in both dementias. In particular, depression was the first symptom that, in bvFTD, a sample could already be observed before the onset of the disease (the mean of the year of the onset of depression is 0.5 year before the onset of cognitive sympthoms). Many studies explored the temporal relationship between depressive symptoms and cognitive impairment (52, 53), suggesting that depression could be able to predict dementia and that mood disorders may be an important “red flag” in detection of neurocognitive impairment and people should learn to recognize them to reach a prompt diagnosis. This may explain why, in our patients, the time elapsed between the onset of symptoms of dementia, and the first visit was delayed (around 4 years). The diagnostic delay (54) may be due to the fact that initial signs of cognitive impairment or deflection of mood in elderly people is considered to be normal, and they may arrive to a clinical consultation only when the concern for their cognitive and behavioral symptoms has become more serious for the patients and/or the families.

This study has potential limitations. Firstly, the different patterns of BPSD that we observed between patients with AD and patients with bvFTD can be related to several genetic risk factors, brain's pathophysiological changes, gender differences, drugs usage, and the different neurobiological profiles of the two pathologies. Indeed, further studies are needed to correlate genetic risk factors with the manifestation of BPSD, as well as to analyze the different patterns of BPSD and brain functional changes across the course of the disease in AD and bvFTD, taking into account gender differences and drugs. Secondly, NPI is a broad-spectrum screening test. In future researches, it may be useful to administer more specific tests for some symptoms, such as geriatric depression scale (GDS) and apathy evaluation scale (AES). Third, several associations appear to be weak, albeit with an alpha value over the significance threshold.

## Conclusion

BPSD profiles of the two groups were similar but not identical. An early and correct identification and evaluation of BPSD are challenges and crucial parts in the clinical approach and management of these disorders. As at the disease onset, BPSD may dominate the clinical picture and may be interpreted as a primary psychiatric disorder, this can delay the proper evaluation and diagnosis of dementia (55). This is an important point as the earlier diagnosis is reached; more families and patients will be able to benefit from the use of appropriate clinical support and possible intervention. Because of their high frequency and impact on management and overall health care resources, BPSD should not be underestimated and be considered as an additional important diagnostic and therapeutic target both in patients with AD and patients with bvFTD. In conclusion, from a qualitative point of view, mood disorders were more often seen in the preclinical phase and/or in the first years of dementias in our cohort, while motor behaviors (aberrant motor activity and wandering) tended to occur in the later stages, generally after 5 years from the onset. This sequence was found in both diseases, and the main difference in BPSD between FTD and AD was quantitative and mainly related to their frequencies, being more represented in FTD than in AD.

## Data Availability Statement

The raw data supporting the conclusions of this article will be made available by the authors, without undue reservation.

## Ethics Statement

Ethical review and approval was not required for the study on human participants in accordance with the local legislation and institutional requirements. The patients/participants provided their written informed consent to participate in this study.

## Author Contributions

VL and FB wrote the manuscript. GP, FF, CC, VL, MM, RC, and SC collected clinical data. VL, FB, NS, GT, GM, DA, AMa, and AMo created database, tables, and figures. NA, NS, and RD performed statistical analysis. VL, GB, RM, and AB conceived and designed the study. All authors revised the manuscript and approved the submitted version.

## Funding

Association for Neurogenetic Research (ARN) provided fund for open access publication fees.

## Conflict of Interest

The authors declare that the research was conducted in the absence of any commercial or financial relationships that could be construed as a potential conflict of interest.

## Publisher's Note

All claims expressed in this article are solely those of the authors and do not necessarily represent those of their affiliated organizations, or those of the publisher, the editors and the reviewers. Any product that may be evaluated in this article, or claim that may be made by its manufacturer, is not guaranteed or endorsed by the publisher.
